# Mortality and Potential Years of Life Lost Attributable to Alcohol Consumption by Race and Sex in the United States in 2005

**DOI:** 10.1371/journal.pone.0051923

**Published:** 2013-01-02

**Authors:** Kevin D. Shield, Gerrit Gmel, Tara Kehoe-Chan, Deborah A. Dawson, Bridget F. Grant, Jürgen Rehm

**Affiliations:** 1 Centre for Addiction and Mental Health, Toronto, Ontario, Canada; 2 Institute of Medical Science, University of Toronto, Toronto, Ontario, Canada; 3 Department of Mathematics, University of Toronto, Toronto, Ontario, Canada; 4 Dalla Lana School of Public Health, University of Toronto, Toronto, Ontario, Canada; 5 Institute for Clinical Psychology and Psychotherapy, Technische Universität Dresden, Dresden, Saxony, Germany; 6 Department of Psychiatry, University of Toronto, Toronto, Ontario, Canada; 7 Laboratory of Epidemiology and Biometry, National Institute on Alcohol Abuse and Alcoholism, National Institutes of Health, Bethesda, Maryland, United States of America; 8 Kelly Government Services, Bethesda, Maryland, United States of America; Indian Institute of Toxicology Research, India

## Abstract

**Background:**

Alcohol has been linked to health disparities between races in the US; however, race-specific alcohol-attributable mortality has never been estimated. The objective of this article is to estimate premature mortality attributable to alcohol in the US in 2005, differentiated by race, age and sex for people 15 to 64 years of age.

**Methods and Findings:**

Mortality attributable to alcohol was estimated based on alcohol-attributable fractions using indicators of exposure from the National Epidemiologic Survey on Alcohol and Related Conditions and risk relations from the Comparative Risk Assessment study. Consumption data were corrected for undercoverage (the observed underreporting of alcohol consumption when using survey as compared to sales data) using adult *per capita* consumption from WHO databases. Mortality data by cause of death were obtained from the US Department of Health and Human Services. For people 15 to 64 years of age in the US in 2005, alcohol was responsible for 55,974 deaths (46,461 for men; 9,513 for women) representing 9.0% of all deaths, and 1,288,700 PYLL (1,087,280 for men; 201,420 for women) representing 10.7% of all PYLL. Per 100,000 people, this represents 29 deaths (29 for White; 40 for Black; 82 for Native Americans; 6 for Asian/Pacific Islander) and 670 PYLL (673 for White; 808 for Black; 1,808 for Native American; 158 for Asian/Pacific Islander). Sensitivity analyses showed a lower but still substantial burden without adjusting for undercoverage.

**Conclusions:**

The burden of mortality attributable to alcohol in the US is unequal among people of different races and between men and women. Racial differences in alcohol consumption and the resulting harms explain in part the observed disparities in the premature mortality burden between races, suggesting the need for interventions for specific subgroups of the population such as Native Americans.

## Introduction

A consistent gap in measures of mortality has been observed for people of different races in the United States (US). The observed large variations by race in measures of mortality have been hypothesized to be attributable in part to differences in exposure to modifiable risk factors, such as alcohol consumption, tobacco smoking, and being overweight or obese [1]. In particular, alcohol consumption has been hypothesized to contribute substantially to this disparity, as volume and patterns of alcohol consumption have been shown to vary greatly by race [2].

Alcohol use is a major risk factor for mortality, with more than 30 International Classification of Diseases (ICD)-10 three digit codes containing alcohol in their name, and more than 200 ICD-10 three-digit codes where alcohol is a component cause [3], [4]. Currently, alcohol is the eighth leading cause of mortality globally, and in 2004 alcohol was responsible for 2.3 million deaths, representing 3.8% of all deaths [4]. For the US in 2005 alcohol consumption has been estimated to be responsible for 64,000 deaths (approximately 45,000 for men and 20,000 for women) for all ages (however, this estimate is based on out of date methodology) [5]. To lessen the burden of disease and injury attributable to alcohol, a global strategy to reduce the harmful use of alcohol was agreed upon at the World Health Organization’s (WHO) 63rd World Health Assembly [6]. A key part of this strategy is to accurately monitor alcohol consumption and the resulting attributable harms, as reliable health data are the foundation of health policies, strategies, and evaluation. Despite the magnitude of mortality attributable to alcohol consumption, alcohol-attributable burden estimates have yet to be calculated by race for any country.

Previous estimates of the burden of mortality attributable to alcohol in the US were limited by being differentiated by sex but not by race [7]–[9], used older and less accurate methods of estimating the burden of mortality [3], and did not include data for alcohol-related diseases such as colon cancer, rectal cancer, and tuberculosis [7]–[9]. Thus, up-to-date, accurate estimates of the alcohol-attributable burden of disease in the US differentiated by race, age and sex are necessary to formulate effective policies and programs to ameliorate health inequalities and to identify cost-effective health interventions that would make the biggest difference to those in the worst health [10], [11].

Accordingly, this paper provides an updated estimate of premature mortality, i.e., the number of deaths and Potential Years of Life Lost (PYLL) below age 65 years attributable to alcohol consumption for the US in 2005 differentiated by race, age and sex.

## Methods

To estimate the number of deaths and PYLL attributable to alcohol consumption, we restricted our analysis to people aged 15 to 64 years for two reasons. First, premature mortality is the most important indicator for public health, as most of the PYLLs can be found in this age category. Second, deaths that occur after the age of 64 are often complicated in pathology and often are miscoded [12], thus contributing to potential bias in comparisons over the full life span. The cut-off of 65 years of age has also been used in other studies that calculate the burden of disease attributable to alcohol consumption [13]–[16]. When comparing alcohol-attributable harms with harms caused by other risk factors based on published studies, we made sure that the same age categories are used.

### Calculation of the Alcohol-Attributable Fractions by Race, Age and Sex

The number of deaths caused by alcohol consumption was calculated using an Alcohol-Attributable Fraction (AAF), which is defined as the fraction of mortality that would not be present if exposure to alcohol was 0 (in this case, if every person was a lifetime abstainer) [17], [18].

The method to calculate the number of deaths and PYLL attributable to alcohol consumption has two main steps: (1) calculation of the race-, age-, sex-, and consumption-specific AAFs, and then (2) application of these AAFs to the corresponding mortality and PYLL dataz.

### Step 1: Calculation of the AAFs by Race, Age and Sex, and of the Alcohol Consumption Exposure Estimates

Alcohol consumption, measured in grams per day, was obtained from the Wave 1 2001–2002 National Epidemiologic Survey on Alcohol and Related Conditions (NESARC 2001–2002) [2]. NESARC 2001–2002 employed a complex, multi-stage sampling that oversampled 18 to 24 year olds, Blacks and Hispanics. Computer-assisted personal interviews were conducted in face-to-face household settings. Individuals living in non-institutionalized group housing, such as military personnel living off base, and residents of institutionalized group housing, such as boarding houses, shelters, and dormitories, were also recruited [19]. In total, 43,093 respondents aged 18 years and older were recruited, with an overall response rate for the survey of 81%.

Summed across estimates for four separate beverage types, volume was estimated based on overall frequency of drinking, usual and largest quantities consumed, frequency of consuming the largest quantity, frequency of consuming 5+ drinks, usual drink size, and ethanol content by volume of the brand usually consumed. The test-retest reliabilities for the various measures of alcohol consumption from the NESARC 2001–2002 were good to excellent, with intra-class correlation coefficients ranging from 0.68 to 0.84 [20]. A coverage rate of 53% between the NESARC 2001–2002 survey and adult *per capita* alcohol consumption for the US was found (estimated as 8.75 liters *per capita* for 2001/2002, the years during which sampling took place), based on the Global Information System for Alcohol and Health (GISAH) database [21]. This means that the survey data combined underestimated the sales and unrecorded data of the US by 47% for 2001/2002. This underestimate is partly due to sampling (some high consuming groups such as the homeless are not part of surveys [22]) or is due to underestimation of one’s own drinking [23].

Consumption estimates were calculated based on race, age and sex. Race in the NESARC 2001–2002 was defined by four categories 1) White (including most Hispanics), 2) Black, 3) Asian/Pacific Islander, and 4) Native American (see (US Census Bureau 1991) for definitions). For individuals who did not identify their race or who identified themselves as multi-racial, the US Census Bureau used an algorithm to impute/assign a race. To correct for undercoverage (the observed underreporting of alcohol consumption when using survey data as compared to adult *per capita* consumption) alcohol consumption data was then triangulated with *per capita* consumption data for 2005 obtained from the GISAH database [16] (for definition and description of coverage rate see [23], [24]). This method of triangulation is detailed elsewhere [25], [26]. A detailed outline of the methods used to model alcohol consumption is outlined in Appendix S1.

### Risk Relations

Sources for Relative Risk (RR) functions by ICD-10 code are outlined in Table S1 in Appendix S2. The RRs, in most cases, were obtained from meta-analyses reporting a continuous RR function by dose of exposure, i.e., by average daily grams of ethanol consumed. In the underlying meta-analyses, older ICD codes were transferred into ICD-10 via the algorithms of Global Burden of Disease study. An outline of the causal relationship between alcohol and these ICD-10 code categories is described in detail elsewhere [3].

### AAF Calculations

For most chronic diseases associated with alcohol consumption, we estimated the number of alcohol-attributable fatalities by combining prevalence data on drinking status, average daily volume of alcohol consumed, and the RR estimates associated with the respective exposure category [3]. In order to estimate the number of fatalities from ischemic heart disease, we combined prevalence data on drinking status, binge drinking status, and average daily volume of alcohol consumed [27], [28]. For alcohol-attributable HIV deaths, we could only estimate the effect of alcohol consumption on adherence to antiretroviral medication [29]. For injuries, volumes of consumption from both average drinking and heavy drinking occasions and frequency of consuming 5 standard drinks or more were combined with the respective RR functions to estimate fatalities (see Appendix S3 for additional details) [30], [31].

### Step 2: Application of the AAFs to Race-, Age- and Sex-specific Mortality, and to PYLL Data Estimates of Mortality

For our analysis, we used both incidence-based (mortality) and time-based (PYLL) measures of public health. Mortality data were obtained from the US Department of Health and Human Services for 2005 by ICD-10 codes, differentiated by age categories, race, and sex. Life expectancies for these age categories were also obtained from the US Department of Health and Human Services by sex for 2005 [32]. ICD-10 codes have been used since 1999 by the US Department of Health and Human Services to report mortality in the United States; however, estimates of deaths prior to 1999 by ICD-10 code are available from the US Department of Health and Human Services [33]. PYLL were calculated using a time-discounting (3%) methodology [34]. Time-discounting methods were used to account for peoples’ preference of a healthy year now, rather than a healthy year in the future. Population estimates for 2005 were based on the latest revisions from the US Census Bureau [2].

### Standardized Rates of Mortality

To directly compare the number of deaths across races while controlling for any differences in age and sex structure of the White, Black, Native American and Asian/Pacific Islander populations, we calculated standardized rates of mortality per 100,000 people using the 2005 US population as the standard population.

All statistics and analyses were performed using R and STATA [35], [36].

## Results

Table 1 outlines the prevalence of “current drinkers,” “former drinkers,” and “lifetime abstainers,” and levels of consumption among current drinkers. In general, average daily consumption, frequency of binge drinking (data not shown), and number of drinks per binge drinking occasion (data not shown) were highest in the youngest age categories, and among men in all age categories. With the exception of Black men, who had a higher average daily consumption than did Native American men, Native Americans had the highest daily consumption, frequency of binge drinking, and number of drinks per binge drinking occasion.

**Table 1:** Key alcohol consumption indicators for the United States for 2005 by race, age and sex.

		Men						Women					
				Prevalence of average alcohol consumption						Prevalence of average alcohol consumption			
Race	Age	Lifetime abstainers	Former drinkers	>0–<40 grams	40 to <60 grams	60 to <100 grams	100+ grams	Lifetime abstainers	Former drinkers	>0–<20 grams	20 to <40 grams	40 to <60 grams	60+ grams
White	15 to 24	16.25%	7.55%	46.41%	10.18%	12.18%	7.43%	21.79%	7.17%	48.96%	12.69%	5.23%	4.16%
	25 to 34	7.85%	9.13%	62.00%	9.41%	8.38%	3.23%	13.97%	11.33%	61.58%	9.65%	2.50%	0.98%
	35 to 44	7.82%	14.73%	56.74%	9.01%	8.31%	3.38%	13.66%	14.74%	58.43%	9.54%	2.57%	1.06%
	45 to 54	8.43%	16.35%	55.57%	8.66%	7.87%	3.13%	14.86%	19.37%	52.21%	9.45%	2.79%	1.32%
	55 to 64	9.10%	21.73%	54.43%	7.16%	5.72%	1.85%	20.33%	21.70%	47.00%	7.87%	2.17%	0.92%
	65+	13.07%	30.10%	47.06%	5.19%	3.63%	0.95%	33.45%	26.50%	34.93%	4.09%	0.81%	0.22%
	Total	10.43%	16.04%	53.73%	8.41%	7.91%	3.48%	19.91%	17.02%	50.22%	8.79%	2.64%	1.42%
Black	15 to 24	23.46%	6.25%	45.75%	9.10%	10.04%	5.41%	36.67%	11.40%	32.50%	9.87%	4.70%	4.86%
	25 to 34	16.73%	10.81%	51.71%	8.70%	8.39%	3.66%	25.82%	14.92%	42.44%	10.17%	3.90%	2.75%
	35 to 44	13.48%	19.07%	46.03%	8.44%	8.73%	4.25%	27.70%	21.32%	33.81%	9.39%	4.12%	3.67%
	45 to 54	12.46%	27.01%	32.32%	8.32%	11.37%	8.53%	25.28%	27.08%	24.49%	9.29%	5.43%	8.43%
	55 to 64	15.44%	30.65%	35.06%	6.98%	7.71%	4.16%	30.29%	32.49%	30.49%	4.90%	1.30%	0.53%
	65+	20.61%	42.81%	30.78%	3.18%	2.12%	0.51%	48.40%	35.32%	13.18%	2.22%	0.62%	0.26%
	Total	17.14%	19.41%	42.02%	7.96%	8.69%	4.79%	31.70%	21.98%	30.57%	8.24%	3.69%	3.82%
Asian/Pacific Islander	15 to 24	24.11%	7.83%	51.24%	7.62%	6.69%	2.51%	42.24%	5.31%	31.41%	10.12%	5.09%	5.83%
	25 to 34	24.02%	7.18%	59.97%	5.19%	3.05%	0.60%	39.72%	16.36%	39.85%	3.46%	0.52%	0.10%
	35 to 44	31.29%	11.56%	44.74%	5.98%	4.83%	1.59%	49.05%	10.81%	39.74%	0.40%	0.01%	0.00%
	45 to 54	13.79%	22.91%	50.42%	6.39%	4.96%	1.53%	55.74%	14.27%	29.43%	0.54%	0.02%	0.00%
	55 to 64	43.82%	7.85%	47.41%	0.77%	0.15%	0.00%	55.99%	15.80%	28.21%	0.00%	0.00%	0.00%
	65+	32.60%	17.87%	46.59%	2.08%	0.78%	0.07%	64.79%	22.43%	12.61%	0.17%	0.00%	0.00%
	Total	26.77%	12.14%	50.59%	5.28%	3.97%	1.25%	49.84%	13.49%	31.86%	2.71%	1.03%	1.07%
Native American	15 to 24	20.63%	9.34%	38.38%	9.60%	12.80%	9.25%	28.66%	6.51%	27.35%	12.09%	8.14%	17.25%
	25 to 34	10.03%	14.64%	59.37%	7.78%	6.19%	1.99%	25.88%	18.75%	50.97%	3.81%	0.50%	0.08%
	35 to 44	13.29%	14.25%	45.68%	9.54%	10.96%	6.28%	13.68%	30.77%	37.36%	10.13%	4.35%	3.72%
	45 to 54	10.46%	25.31%	44.09%	8.00%	8.20%	3.94%	30.66%	25.82%	33.15%	6.82%	2.27%	1.28%
	55 to 64	7.55%	41.83%	37.77%	5.75%	5.13%	1.98%	21.40%	28.96%	46.06%	3.14%	0.38%	0.06%
	65+	18.91%	34.13%	45.08%	1.42%	0.43%	0.03%	45.81%	28.45%	15.99%	4.91%	2.36%	2.49%
	Total	14.05%	19.08%	45.55%	7.89%	8.57%	4.87%	26.86%	21.24%	35.48%	7.52%	3.54%	5.37%

**Figure 1:**
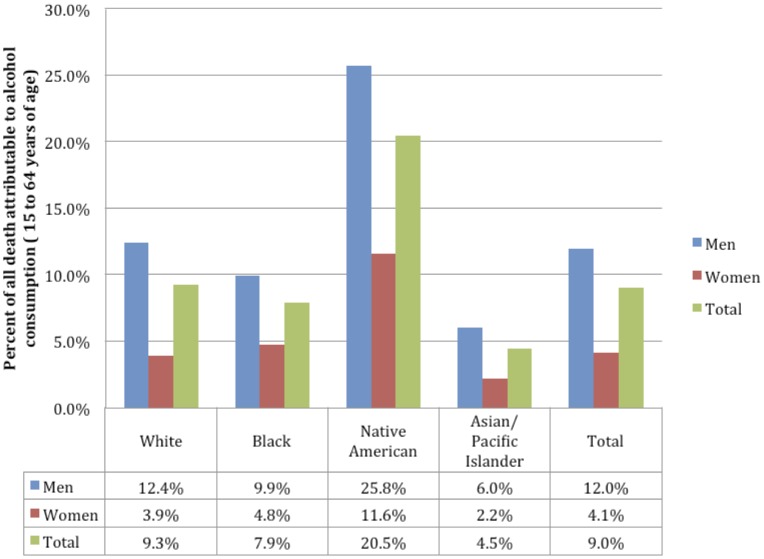
Percentage of all deaths attributable to alcohol consumption by race and sex for people aged 15 to 64 years.

For people aged 15 to 64 years, alcohol was responsible for 55,974 deaths in 2005 representing 9.0% of all deaths in that age range. This number can be broken down by sex into 46,461 representing 12.0% of all deaths of men, and 9,513 representing 4.1% of all deaths of women (see Table 2 for the number of deaths attributable to alcohol by sex and by cause, Figure 1 for the percentage of deaths attributable to alcohol consumption by sex and race, and Appendix S4 for the percentage of deaths attributable to alcohol consumption by major causes of death). These are net deaths, where the beneficial effects of alcohol, such as the protective effects of low to moderate drinking on ischemic disease and diabetes, have already been subtracted.

**Table 2:** Deaths attributable to alcohol consumption by cause, race, age and sex for the US in 2005.

	Men						Women						Total
Race	15 to 24	25 to 34	35 to 44	45 to 54	55 to 64	15 to 64	15 to 24	25 to 34	35 to 44	45 to 54	55 to 64	15 to 64	
Total													
All causes of death (total)	25,509	29,283	53,309	114,472	165,429	388,002	8,725	12,642	31,476	69,058	109,872	231,773	619,775
All causes of death (alcohol attributable)	10,995	7,785	10,044	11,444	6,193	46,461	1,030	747	1,876	3,729	2,131	9,513	55,974
All causes of death (% alcohol attributable)	43.1%	26.6%	18.8%	10.0%	3.7%	12.0%	11.8%	5.9%	6.0%	5.4%	1.9%	4.1%	9.0%
White													
All causes of death (total)	18,792	21,232	40,824	88,882	134,272	304,002	6,618	8,871	22,760	51,342	87,946	177,537	481,539
All causes of death (alcohol attributable)	9626	6616	8654	8228	4621	37,745	835	556	1364	2374	1760	6,889	44,634
All causes of death (% alcohol attributable)	51.2%	31.2%	21.2%	9.3%	3.4%	12.4%	12.6%	6.3%	6.0%	4.6%	2.0%	3.9%	9.3%
Communicable, maternal, perinatal, and nutritional conditions													
Infectious and parasitic diseases	2	2	6	10	9	29	1	0	1	1	1	4	33
HIV/AIDS	1	8	37	36	12	94	0	3	8	7	2	20	114
Respiratory infections	13	16	46	104	143	322	4	9	21	48	75	157	479
Noncommunicable diseases													
Malignant neoplasms	10	32	206	1,054	1,650	2,952	4	37	252	709	1,025	2,027	4,979
Diabetes	−1	−7	−22	−61	−130	−221	−9	−27	−92	−244	−465	−837	−1,058
Neuro-psychiatric conditions	95	193	870	1,755	1,395	4,308	17	57	234	527	370	1,205	5,513
Cardiovascular disease	27	14	29	−650	−1,710	−2,290	14	4	58	132	18	226	−2,064
Digestive diseases	10	100	773	2,030	1,584	4,497	3	36	342	703	484	1,568	6,065
Injuries													
Unintentional injuries	7,569	4,894	5,010	3,213	1,378	22,064	733	393	469	411	217	2,223	24,287
Intentional injuries	1,900	1,362	1,696	733	287	5,978	67	42	68	77	33	287	6,265
Underdetermined intent	0	2	3	4	3	12	1	2	3	3	0	9	21
Black													
All causes of death (total)	5,709	6,843	10,626	22,248	26,711	72,137	1,662	3,193	7,560	15,501	18,705	46,621	118,758
All causes of death (alcohol attributable)	930	936	1037	2853	1421	7,177	112	150	409	1240	314	2,225	9,402
All causes of death (% alcohol attributable)	16.3%	13.7%	9.8%	12.8%	5.3%	9.9%	6.7%	4.7%	5.4%	8.0%	1.7%	4.8%	7.9%
Communicable, maternal, perinatal, and nutritional conditions													
Infectious and parasitic diseases	1	1	3	6	6	17	0	1	0	2	1	4	21
HIV/AIDS	1	10	31	34	12	88	1	7	14	12	3	37	125
Respiratory infections	3	5	16	46	39	109	1	3	7	21	12	44	153
Noncommunicable diseases													
Malignant neoplasms	4	11	53	325	396	789	3	18	98	336	168	623	1,412
Diabetes	−1	−2	−4	7	−4	−4	−3	−12	−24	−44	−89	−172	−176
Neuro-psychiatric conditions	11	30	116	295	233	685	7	9	46	87	55	204	889
Cardiovascular disease	10	26	104	305	93	538	9	26	128	561	67	791	1,329
Digestive diseases	4	9	78	295	196	582	0	7	51	121	58	237	819
Injuries													
Unintentional injuries	750	707	571	1,373	419	3,820	88	79	82	134	38	421	4,241
Intentional injuries	146	139	68	167	31	551	6	12	6	10	1	35	586
Underdetermined intent	1	0	1	0	0	2	0	0	1	0	0	1	3
Native American													
All causes of death (total)	439	512	777	1,160	1,341	4,229	196	208	446	767	899	2,516	6,745
All causes of death (alcohol attributable)	268	167	278	255	119	1,087	64	30	87	87	22	290	1,377
All causes of death (% alcohol attributable)	61.0%	32.6%	35.8%	22.0%	8.9%	25.7%	32.7%	14.4%	19.5%	11.3%	2.4%	11.5%	20.4%
Communicable, maternal, perinatal, and nutritional conditions													
Infectious and parasitic diseases	0	0	1	0	0	1	0	0	0	0	0	0	1
HIV/AIDS	0	0	0	0	0	0	0	0	0	0	0	0	0
Respiratory infections	0	0	1	1	2	4	0	0	0	1	1	2	6
Noncommunicable diseases													
Malignant neoplasms	0	0	4	9	13	26	0	0	2	5	4	11	37
Diabetes	0	0	0	−1	−1	−2	0	−1	−2	−3	−8	−14	−16
Neuro-psychiatric conditions	5	6	47	61	28	147	3	3	22	36	7	71	218
Cardiovascular disease	1	1	5	−1	−8	−2	1	0	4	5	−2	8	6
Digestive diseases	1	12	46	63	41	163	0	6	33	32	16	87	250
Injuries													
Unintentional injuries	183	123	135	103	41	585	44	20	25	11	4	104	689
Intentional injuries	77	25	38	19	3	162	16	2	3	0	0	21	183
Underdetermined intent	1	0	1	1	0	3	0	0	0	0	0	0	3
Asian/Pacific Islander													
All causes of death (total)	569	696	1,082	2,182	3,105	7,634	249	370	710	1,448	2,322	5,099	12,733
All causes of death (alcohol attributable)	171	66	75	108	32	452	19	11	16	28	35	109	561
All causes of death (% alcohol attributable)	30.1%	9.5%	6.9%	4.9%	1.0%	5.9%	7.6%	3.0%	2.3%	1.9%	1.5%	2.1%	4.4%
Communicable, maternal, perinatal, and nutritional conditions													
Infectious and parasitic diseases	1	0	0	0	0	1	0	0	0	0	0	0	1
HIV/AIDS	0	0	0	0	0	0	0	0	0	0	0	0	0
Respiratory infections	0	0	0	2	1	3	0	0	0	0	1	1	4
Noncommunicable diseases													
Malignant neoplasms	1	3	13	34	18	69	0	2	7	15	18	42	111
Diabetes	0	0	0	−2	−3	−5	0	0	−1	−1	−1	−3	−8
Neuro-psychiatric conditions	1	11	12	19	12	55	0	1	3	3	1	8	63
Cardiovascular disease	0	−2	4	−5	−21	−24	1	1	1	3	9	15	−9
Digestive diseases	0	3	13	25	4	45	0	2	2	3	3	10	55
Injuries													
Unintentional injuries	140	37	27	29	18	251	15	4	4	4	4	31	282
Intentional injuries	28	14	6	6	3	57	3	1	0	0	0	4	61
Underdetermined intent	0	0	0	0	0	0	0	0	0	1	0	1	1

Most of the deaths attributable to alcohol were due to injuries, with 36,622 such deaths (33,485 for men; 3,137 for women). In terms of the number of deaths attributable to alcohol, the net effect was highest for the older age groups, peaking in the age range of 45 to 54 years; however, this was dependent on the cause of death, with people in younger age categories experiencing much more mortality attributable to injuries than was experienced by people in older age groups.

**Figure 2:**
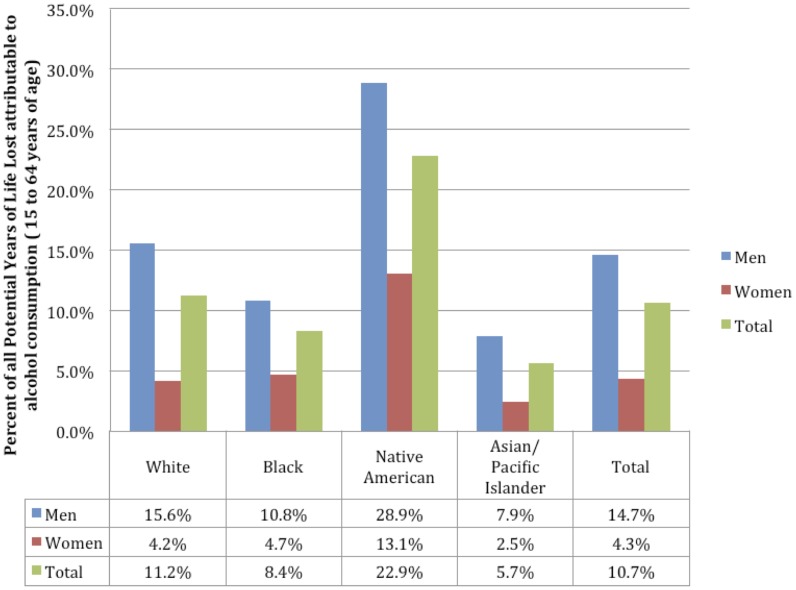
Percentage of all Potential Years of Life Lost attributable to alcohol consumption by race and sex for people aged 15 to 64 years.

In the US in 2005 for people aged 15 to 64 years, 1,288,700 PYLL, representing 10.7% of all PYLL, were attributable to alcohol. This number can be broken down by sex into 1,087,280 representing 14.7% of all PYLL for men, and 201,420 PYLL representing 4.3% of all PYLL for women (see Table 3 for the number of PYLL attributable to alcohol by race, age, sex and cause, and Figure 2 for the percentage of PYLL attributable to alcohol consumption by sex and race). As with mortality, the biggest contributor to PYLL attributable to alcohol was injuries, which constituted 7.2% of all PYLL (10.6% for men and 1.6% for women) for people aged 15 to 64 years. Overall, alcohol was responsible for a greater percentage of all PYLL for men when compared to women. Native Americans had the highest percentage of PYLL attributable to alcohol consumption (22.8%) when compared to any other group, with people who identified as White having the second highest percentage of PYLL attributable to alcohol consumption (11.2%).

**Table 3:** Potential Years of Life Lost attributable to alcohol consumption by cause, race, age and sex for the US in 2005.

	Men						Women						Total
Race	15 to 24	25 to 34	35 to 44	45 to 54	55 to 64	15 to 64	15 to 24	25 to 34	35 to 44	45 to 54	55 to 64	15 to 64	
Total													
All causes of death (total)	682,340	738,580	1,205,700	2,217,950	2,572,180	7,416,750	240,970	331,550	750,970	1,439,190	1,881,150	4,643,830	12,060,580
All causes of death (alcohol attributable)	295,060	205,500	283,470	213,170	90,080	1,087,280	32,620	18,510	42,500	71,050	36,740	201,420	1,288,700
All causes of death (% alcohol attributable)	43.2%	27.8%	23.5%	9.6%	3.5%	14.7%	13.5%	5.6%	5.7%	4.9%	2.0%	4.3%	10.7%
White													
All causes of death (total)	502,670	535,520	923,330	1,722,130	2,087,730	5,771,380	182,780	232,650	543,020	1,069,980	1,505,750	3,534,180	9,305,560
All causes of death (alcohol attributable)	258110	172750	245350	154590	67680	898,480	25570	12420	31400	47150	30170	146,710	1,045,190
All causes of death (% alcohol attributable)	51.3%	32.3%	26.6%	9.0%	3.2%	15.6%	14.0%	5.3%	5.8%	4.4%	2.0%	4.2%	11.2%
Communicable, maternal, perinatal, and nutritional conditions													
Infectious and parasitic diseases	60	60	130	200	140	590	10	10	20	20	20	80	670
HIV/AIDS	20	210	840	710	190	1,970	10	70	180	140	30	430	2,400
Respiratory infections	350	400	1,030	2,010	2,230	6,020	100	220	490	990	1,290	3,090	9,110
Noncommunicable diseases													
Malignant neoplasms	270	800	4,660	20,420	25,650	51,800	110	980	6,020	14,780	17,540	39,430	91,230
Diabetes	−20	−180	−500	−1,190	−2,020	−3,910	−240	−720	−2,200	−5,080	−7,960	−16,200	−20,110
Neuro-psychiatric conditions	2,540	4,870	19,670	34,010	21,690	82,780	470	1,510	5,580	10,990	6,340	24,890	107,670
Cardiovascular disease	1,340	6,230	50,320	−17,430	−30,770	9,690	2,920	−2,040	260	420	350	1,910	11,600
Digestive diseases	260	2,520	17,470	39,320	24,630	84,200	70	930	8,170	14,660	8,280	32,110	116,310
Injuries													
Unintentional injuries	202,460	123,430	113,310	62,260	21,430	522,890	20,230	10,300	11,200	8,560	3,710	54,000	576,890
Intentional injuries	50,830	34,360	38,350	14,200	4,460	142,200	1,860	1,110	1,610	1,610	570	6,760	148,960
Underdetermined intent	0	50	70	80	50	250	30	50	70	60	0	210	460
Black													
All causes of death (total)	152,710	172,600	240,330	431,060	415,320	1,412,020	45,900	83,740	180,370	323,050	320,250	953,310	2,365,330
All causes of death (alcohol attributable)	25170	26560	28770	51820	20110	152,430	4350	5040	8640	21590	5560	45,180	197,610
All causes of death (% alcohol attributable)	16.5%	15.4%	12.0%	12.0%	4.8%	10.8%	9.5%	6.0%	4.8%	6.7%	1.7%	4.7%	8.4%
Communicable, maternal, perinatal, and nutritional conditions													
Infectious and parasitic diseases	40	20	60	110	90	320	10	20	0	40	10	80	400
HIV/AIDS	30	250	700	650	190	1,820	20	190	330	250	50	840	2,660
Respiratory infections	70	120	350	880	610	2,030	20	70	170	430	210	900	2,930
Noncommunicable diseases													
Malignant neoplasms	110	290	1,200	6,300	6,160	14,060	80	460	2,330	7,000	2,870	12,740	26,800
Diabetes	−20	−60	−90	140	−60	−90	−100	−320	−580	−910	−1,530	−3,440	−3,530
Neuro-psychiatric conditions	300	750	2,620	5,710	3,620	13,000	210	230	1,100	1,820	940	4,300	17,300
Cardiovascular disease	550	3,630	7,690	2,480	−550	13,800	1,520	1,820	1,950	7,460	1,350	14,100	27,900
Digestive diseases	110	220	1,760	5,710	3,050	10,850	10	190	1,220	2,510	1,000	4,930	15,780
Injuries													
Unintentional injuries	20,060	17,830	12,910	26,600	6,520	83,920	2,430	2,080	1,950	2,790	640	9,890	93,810
Intentional injuries	3,890	3,510	1,550	3,240	480	12,670	150	300	150	200	20	820	13,490
Underdetermined intent	30	0	20	0	0	50	0	0	20	0	0	20	70
Native American													
All causes of death (total)	11,740	12,910	17,570	22,480	20,850	85,550	5,410	5,460	10,640	15,980	15,390	52,880	138,430
All causes of death (alcohol attributable)	7150	4280	6550	4900	1810	24,690	1910	780	2080	1750	400	6,920	31,610
All causes of death (% alcohol attributable)	60.9%	33.2%	37.3%	21.8%	8.7%	28.9%	35.3%	14.3%	19.5%	11.0%	2.6%	13.1%	22.8%
Communicable, maternal, perinatal, and nutritional conditions													
Infectious and parasitic diseases	0	10	20	10	10	50	0	0	0	0	0	0	50
HIV/AIDS	0	0	10	10	0	20	0	0	10	0	0	10	30
Respiratory infections	0	10	20	30	30	90	0	0	10	10	10	30	120
Noncommunicable diseases													
Malignant neoplasms	0	0	90	170	200	460	0	10	60	100	70	240	700
Diabetes	0	−10	0	−10	−20	−40	0	−20	−40	−70	−140	−270	−310
Neuro-psychiatric conditions	130	150	1,070	1,180	440	2,970	90	80	530	760	120	1,580	4,550
Cardiovascular disease	20	70	380	−70	−170	230	180	−30	40	50	−10	230	460
Digestive diseases	20	300	1,030	1,210	630	3,190	0	150	800	660	280	1,890	5,080
Injuries													
Unintentional injuries	4,900	3,110	3,060	1,990	640	13,700	1,210	540	600	230	70	2,650	16,350
Intentional injuries	2,050	640	850	360	50	3,950	430	50	70	10	0	560	4,510
Underdetermined intent	30	0	20	20	0	70	0	0	0	0	0	0	70
Asian/Pacific Islander													
All causes of death (total)	15,220	17,550	24,470	42,280	48,280	147,800	6,880	9,700	16,940	30,180	39,760	103,460	251,260
All causes of death (alcohol attributable)	4630	1910	2800	1860	480	11,680	790	270	380	560	610	2,610	14,290
All causes of death (% alcohol attributable)	30.4%	10.9%	11.4%	4.4%	1.0%	7.9%	11.5%	2.8%	2.2%	1.9%	1.5%	2.5%	5.7%
Communicable, maternal, perinatal, and nutritional conditions													
Infectious and parasitic diseases	10	10	0	10	10	40	10	0	0	10	0	20	60
HIV/AIDS	0	0	10	10	0	20	0	0	0	0	0	0	20
Respiratory infections	10	10	10	40	20	90	0	10	10	10	20	50	140
Noncommunicable diseases													
Malignant neoplasms	40	70	280	660	280	1,330	0	50	160	320	310	840	2,170
Diabetes	0	−10	−10	−30	−50	−100	0	−10	−20	−30	−10	−70	−170
Neuro-psychiatric conditions	30	290	280	370	190	1,160	0	30	70	60	20	180	1,340
Cardiovascular disease	40	170	1,170	−370	−370	640	280	20	−10	30	150	470	1,110
Digestive diseases	0	80	300	490	70	940	10	50	60	60	50	230	1,170
Injuries													
Unintentional injuries	3,760	930	610	560	280	6,140	420	100	100	80	70	770	6,910
Intentional injuries	740	360	150	120	50	1,420	70	20	10	0	0	100	1,520
Underdetermined intent	0	0	0	0	0	0	0	0	0	20	0	20	20

**Figure 3:**
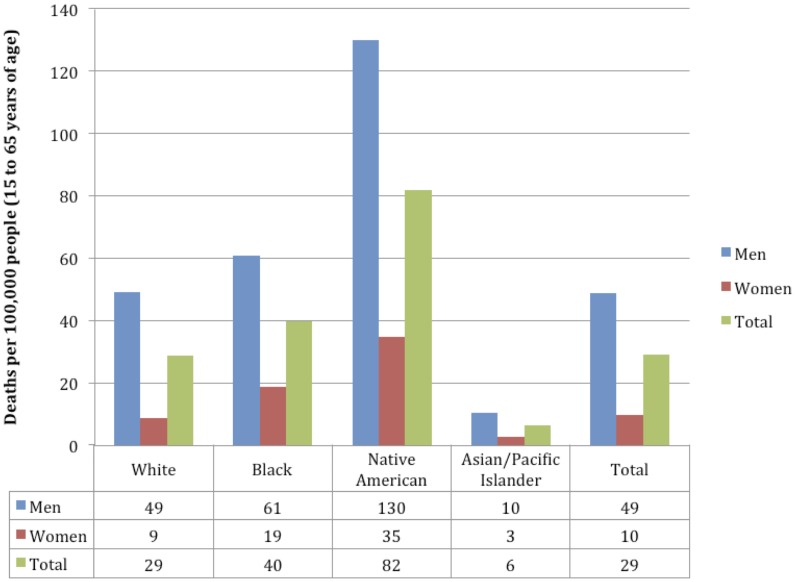
Population standardized deaths per 100,000 people attributable to alcohol consumption by race and sex.

**Figure 4:**
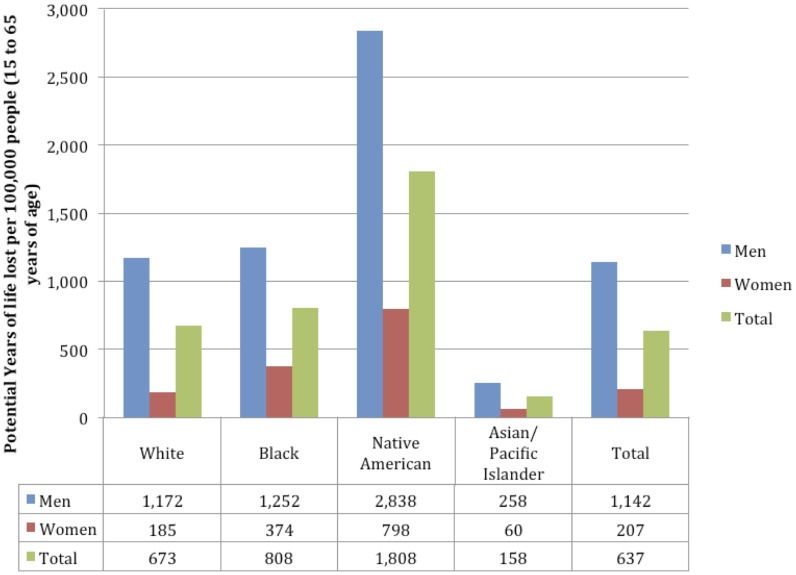
Population standardized Potential Years of Life Lost per 100,000 people attributable to alcohol consumption by race and sex.

In the US in 2005 the standardized rates of alcohol-attributable mortality and PYLL per 100,000 people varied by sex and race (see Figure 3 for population standardized mortality rates and Figure 4 for population standardized PYLL rates, in each case for 2005), reflecting a difference in both drinking patterns and causes of mortality while controlling for population structure. Men experienced 49 alcohol-attributable deaths and 1,142 alcohol-attributable PYLL and women experienced 10 alcohol-attributable deaths and 207 alcohol-attributable PYLL, in each case per 100,000 people. In terms of race, Native Americans experienced many more harms for both sexes, with 82 deaths per 100,000 people (130 for men; 35 for women) and 1,808 PYLL per 100,000 people (2,838 for men; 798 for women). This rate of mortality for Native Americans represented more than ten times the rate experienced by Asian/Pacific Islanders. Asian/Pacific Islanders experienced the lowest rates of harms for both mortality and PYLL. In terms of the two largest populations in the US, people who identified themselves as White experienced fewer harms in terms of mortality and PYLL per 100,000 people when compared to people who identified as Black; people who identified themselves as White experienced 29 deaths (49 for men; 9 for women) and 673 PYLL (1,172 for men; 185 for women) per 100,000, and people who identified as Black experienced 40 deaths (61 for men; 19 for women) and 808 PYLL (1,252 for men; 374 for women) per 100,000.

## Comment

This is the first report which outlines the burden of mortality and PYLL attributable to alcohol consumption in the US differentiated by race, age and sex, and it reveals that alcohol consumption is a large contributor to the burden of mortality. As previously hypothesized, this variation in premature mortality provides evidence that a health disparity in alcohol-attributable harms exists across races in the US. In particular, Native Americans, and to a lesser extent Black Americans, when compared to other races, have a higher standardized rate of alcohol-attributable mortality and PYLL. Thus, alcohol consumption can be seen as a main contributor underlying the known health disparities in the US [1]. In addition to racial differences in alcohol-attributable mortality, men in every racial group experienced more than three times the amount of mortality when compared to women.

The observed differences in alcohol-attributable mortality across races may not be attributable to alcohol consumption alone as health-care utilization and the underlying population risk for alcohol-related diseases, injuries and conditions also impact on differences in the burden of alcohol-attributable mortality across races [17], [18]. Thus, effective interventions and policies aimed at addressing this disparity should address race differences in health plan coverage and health-care utilization for alcohol-related conditions [1], and should include race-specific interventions aimed at reducing the volume of alcohol consumed and deterring harmful alcohol consumption patterns [37], [38].

This analysis has certain limitations, such as the quality of health outcomes data [39]. Information concerning cause of death has long been seen as containing inaccuracies [40], and more recent studies still confirm considerable degrees of error in such information [41], [42]. Additionally, the exposure estimates for drinking status and binge drinking patterns used in our analysis were measured in 2001, whereas the outcomes were measured in 2005; however, the length of time between 2001 and 2005 should not greatly affect the alcohol-attributable mortality and PYLL estimates as alcohol consumption in the US remained relatively stable from 2001 to 2005 [43]. The estimates of alcohol consumption used in our analysis were also cross-sectional, i.e., measured more or less concurrently with deaths and PYLL, whereas long-term patterns of alcohol consumption impact the risk of some chronic diseases such as cancer [44].

Furthermore, our analysis did not include all aspects of harms to others (such as motor vehicle accidents, and assaults), which recently have been shown to constitute a large proportion of the burden of injury attributable to alcohol [45]; this exclusion was due to an absence of a methodology to calculate these harms by race, age and sex [15].

We also did not estimate the number of deaths for people in the US over the age of 64 due to the unreliability of data relating to cause of death in the elderly [12]. If the age group above 64 years had been included in our analysis, we estimated that the number of deaths and PYLL attributable to alcohol would have increased from 55,974 deaths and 1,288,700 PYLL to 82,213 deaths and 1,557,030 PYLL. Our analysis focused on premature mortality; however, in a case where all alcohol-attributable mortality is examined, injury may play a smaller part, as its role in causing death in people 65 years of age and older is relatively smaller than its same role in younger age groups [32].

This analysis was based only on race and does not provide alcohol-attributable estimates by ethnicity or socio-economic status. Alcohol consumption has been shown to vary by both ethnicity and socio-economic status [2] and, thus, the alcohol-attributable harms are expected to vary by these variables as well [46]. The exclusion from this paper of analyses of these variables was due to the unavailability of data differentiated by ethnicity and socio-economic status.

This analysis is also based on RR functions that were usually differentiated by sex and adjusted for age and smoking status, and in some cases for a variety of other risk factors. While the use of adjusted RR functions may introduce bias (see [47]–[49]) most of the published literature on risk factors only report adjusted RRs and, thus, they are the basis of almost all comparative risk analyses ([39]; for alcohol see the publications outlined in Appendix S2). For the risk estimates for alcohol in particular, most analyses show no marked differences after adjustment for the usual confounders and effect measure modifiers tested (see [3], and the meta-analyses cited there). However, there may be a need for adjustment to the RRs for alcohol if future research indicates that other dimensions of alcohol consumption, such as irregular heavy drinking occasions, impact the risk estimates.

For our analysis, we corrected the survey estimates of consumption so that the coverage of the alcohol consumption data used was equal to 80% of the US *per capita* consumption for 2005 (the *per capita* consumption of alcohol in 2005 was 9.5 liters of pure alcohol per person). If we had not triangulated the survey data based on total adult *per capita* consumption, the survey coverage rate would have been 49.7% for 2005. If unadjusted alcohol consumption survey data were used to calculate the burden of alcohol consumption, we estimated that alcohol would be responsible for 49,788 deaths and 1,120,740 PYLL for people aged 15 to 64 years. These results are similar to those which we calculated using a coverage rate of 80%, where it was estimated that 55,974 deaths and 1,228,700 PYLL were attributable to alcohol consumption.

The incomplete coverage of *per capita* consumption in the NESARC 2001–2002, typical of survey-based consumption estimates, may have been due to disproportionately high levels of consumption among non-responders and to not capturing in the sampling frame people who were homeless and not living in shelters (about half of all people who are homeless in the US) [50]. This may be a concern as a relatively small proportion of the population is responsible for the majority of the alcohol consumed. For instance, in the NESARC 2001–2002 sample, 6.7% of White male drinkers consumed 33% of the overall consumption, so excluding or undersampling of small groups with high consumption may result in a large degree of undercoverage [51]. However, given that the unsheltered, homeless population represents a small fraction of the total population (0.1% of those people 15 years of age and older), their inclusion in the NESARC 2001–2002 would have increased the survey coverage rates by less than 1% (on the basis of the assumptions in Shield and Rehm [22]). Thus, almost all of the undercoverage results from incomplete reporting of consumption among survey respondents and disproportionately high levels of consumption among the non-responders who were part of the sampling frame.

It should be noted that our analysis did not take into account morbidity attributable to alcohol consumption. As alcohol consumption has a greater impact on morbidity (as measured by Years Lived with Disability (YLD), a metric which combines the duration lived with a disease or injury and the severity of the disease or injury) than on mortality or premature mortality (as measured in PYLL), metrics such as Disability Adjusted Life Years (DALYs) (a measure that combines PYLL and YLD) are required to accurately characterize the burden of alcohol consumption.

Danaei and colleagues estimated that alcohol consumption was responsible for 64,000 deaths (45,000 for men and 20,000 for women) for all ages in the US in 2005 [5]. These estimates are substantially lower than our study’s estimates of 82,213 deaths (61,539 for men and 20,674 for women) for all ages. Our updated estimates of the burden of alcohol consumption show that alcohol is a greater risk factor for mortality in the US than was previously thought. Differences between our estimates and those of Danaei and colleagues may be explained by our use of better modeling methods for alcohol consumption and its associated risks, and our use of alcohol consumption data corrected for undercoverage. In addition, we included alcohol-attributable causes of death not included in the study by Danaei and colleagues, such as infectious diseases [52].

### Comparison to Other Risk Factors

Our updated estimate of the burden of alcohol consumption in terms of mortality for the US is still lower than the burden estimated for tobacco, and poor diet and physical inactivity [53]; tobacco use was responsible for an estimated 435,000 deaths, and poor diet and physical inactivity were responsible for an estimated 400,000 deaths in the US in 2000 (there were no age restrictions used when calculating these estimates.

## Conclusion

This is the first study which compares alcohol-attributable mortality and PYLL across different races in the US. However, since alcohol consumption also has effects on social harms, more research is needed to quantify the alcohol-attributable social harms differentiated by race, age and sex. Since there is a disparity in alcohol-attributable harms in the US between racial groups, research is required to identify the mechanisms that give rise to and sustain these disparities in order to effectively develop and target alcohol policy strategies.

## Supporting Information

Appendix S1 **Alcohol consumption modeling methodology.**
(DOCX)

Appendix S2 **Categories of alcohol-related diseases and sources used for determining alcohol-attributable fractions.**
(DOCX)

Appendix S3 **Alcohol-attributable fraction modeling methodology.**
(DOCX)

Appendix S4 **Alcohol-attributable fractions by major causes of death.**
(DOCX)
