# Supplementary material for: Mortality and Potential Years of Life Lost Attributable to Alcohol Consumption by Race and Sex in the United States in 2005
Source: PLoS One. 2013 Jan 2;8(1):e51923. doi: 10.1371/journal.pone.0051923 (PMC3534703; doi:10.1371/journal.pone.0051923)
Supplement: Appendix S1 — Alcohol consumption modeling methodology. (DOCX) [file pone.0051923.s001.docx]

## Appendix 1 Alcohol consumption modeling methodology

There are two reasons for upshifting survey estimates of alcohol consumption to a level of exposure which is defined by a proportion of *per capita* consumption [1]: 1) it enables comparisons among different countries and populations such as in the global Comparative Risk Assessment (CRA) study (see Rehm et al., 2009 and Rehm et al., 2003 [2,3]), similar to a side-by-side evaluation of standardized rates [4], and 2) it corrects for the underestimation of drinking in survey data, which if uncorrected would result in an underestimation of the burden of disease attributable to alcohol. However, the upshift in survey estimates of alcohol consumption should be to a level representing approximately 80% of *per capita* consumption to account for undercoverage of alcohol consumption in the medical epidemiology studies upon which the RR estimates are based and to account for alcohol sold/produced but not consumed [5,6]. This appendix outlines the upshifting methods used to estimate alcohol consumption in the United States for 2005.

*Modeling alcohol consumption*

By regressing the means (μ) and standard deviations (σ) of over 500 consumption distributions from more than 66 countries (representing mostly high- and middle-income countries), Rehm and colleagues found that the standard deviation could be expressed as a function of the mean as follows [7]:

(Formula 1)

$$\sigma_{men}=1.171*\mu_{men}$$

(Formula 2)

$$\sigma_{women}=1.258*\mu_{women}$$

For our analysis, we triangulated alcohol consumption data obtained from the NESARC 2001-2002 and corrected it for undercoverage with the 2005 U.S. adult *per capita* consumption data. Adult *per capita* consumption of alcohol in the U.S., defined as the average number of liters (l) of pure alcohol consumed by an adult (15 years +) per year, was calculated by adding the estimated recorded and unrecorded adult *per capita* consumption. Adult *per capita* consumption estimates (i.e., recorded and unrecorded consumption) were obtained from the GISAH database [8], and using the procedures of the ongoing CRA study within the 2005 Global Burden of Disease (GBD) study, we averaged the 2004 to 2006 estimates to approximate consumption in 2005.

An analysis of over 851 consumption distributions from numerous countries (mostly high- and middle-income countries) determined that survey data of alcohol consumption were best modeled using a Gamma distribution [7]. In order to model alcohol consumption continuously, we used the above-noted triangulated alcohol consumption data and the relationship between alcohol consumption and the standard deviation of the Gamma distribution as outlined by Rehm and colleagues to calculate the shape and scale parameters of the Gamma distribution of alcohol consumption [7].

Reference List

1. Rehm J, Klotsche J, Patra J (2007) Comparative quantification of alcohol exposure as risk factor for global burden of disease. Int J Methods Psychiatr Res 16: 66-76.

2. Rehm J, Mathers C, Popova S, Thavorncharoensap M, Teerawattananon Y, et al. (2009) Global burden of disease and injury and economic cost attributable to alcohol use and alcohol use disorders. Lancet 373: 2223-2233.

3. Rehm J, Room R, Monteiro M, Gmel G, Graham K, et al. (2003) Alcohol as a risk factor for global burden of disease. Eur Addict Res 9: 157-164.

4. Rothman,KJ, Greenland,S, and Lash,TL (2008) Modern Epidemiology, 3rd ed. Pennsylvania, USA: Lippincott Williams & Wilkins.

5. Shield K, Rehm J (2012) Difficulties with telephone-based surveys on alcohol in high-income countries: the Canadian example. Int J Methods Psychiatr Res 21: 17-28.

6. Gmel G, Rehm J (2004) Measuring alcohol consumption. Contemp Drug Probl 31: 467-540.

7. Rehm J, Kehoe T, Gmel G, Stinson F, Grant B, et al. (2010) Statistical modeling of volume of alcohol exposure for epidemiological studies of population health: the example of the US. Popul Health Metr 8: 3.

8. World Health Organization (2011) Global Information System on Alcohol and Health. Geneva, Switzerland: World Health Organization.
