# Supplementary material for: Mortality and Potential Years of Life Lost Attributable to Alcohol Consumption by Race and Sex in the United States in 2005
Source: PLoS One. 2013 Jan 2;8(1):e51923. doi: 10.1371/journal.pone.0051923 (PMC3534703; doi:10.1371/journal.pone.0051923)
Supplement: Appendix S2 — Categories of alcohol-related diseases and sources used for determining alcohol-attributable fractions. (DOCX) [file pone.0051923.s002.docx]

### Appendix 2: Categories of alcohol-related diseases and sources used for determining alcohol-attributable fractions

| Table S1. Categories of alcohol-related disease and sources used for determining alcohol-attributable fractions | | | |
| --- | --- | --- | --- |
|  |  |  |  |
| Condition | | ICD 10 Code | Source for Relative Risk |
| Infectious and parasitic diseases | |  |  |
|  | Tuberculosis | A15-A19, B90 | Lönnroth et al., 2008 [1] (causal relationship see: Rehm et al., 2009)[2] |
| Malignant neoplasm's | |  |  |
|  | Mouth and oropharynx cancers | C00-C14 | Baan R et al., 2004 [3] (based on Relative risks from Corrao et al., 2004)[4] |
|  | Esophageal cancer | C15 | Baan R et al., 2004 [3] (based on Relative risks from Corrao et al., 2004)[4] |
|  | Liver cancer | C22 | Baan R et al., 2004 [3] (based on Relative risks from Corrao et al., 2004)[4] |
|  | Laryngeal cancer | C32 | Baan R et al., 2004 [3] (based on Relative risks from Corrao et al., 2004)[4] |
|  | Breast cancer | C50 | Baan R et al., 2004 [3] (based on Relative risks from Corrao et al., 2004)[4] |
|  | Colon cancer | C18 | Baan R et al., 2004 [3] (based on Relative risks from Corrao et al., 2004)[4] |
|  | Rectal cancer | C20 | Baan R et al., 2004 [3] (based on Relative risks from Corrao et al., 2004)[4] |
| Diabetes | |  |  |
|  | Diabetes mellitus | E10-E14 | Baliunas et al., 2009 [5] |
| Neuro-psychiatric conditions | |  |  |
|  | Alcoholic psychoses | F10.0, F10.3-F10.9 | 100% AAF per definition |
|  | Alcohol abuse | F10.1 | 100% AAF per definition |
|  | Alcohol dependence syndrome | F10.2 | 100% AAF per definition |
|  | Degeneration of nervous system due to alcohol | G31.2 | 100% AAF per definition |
|  | Epilepsy | G40-G41 | Samokhvalov et al., 2010 [6] |
|  | Alcohol polyneuropathy | G62.1 | 100% AAF per definition |
| Cardiovascular disease | |  |  |
|  | Hypertensive disease | I10-I15 | Taylor et al., 2010 [7] |
|  | Ischemic heart disease | I20-I25 | Roerecke & Rehm 2011 [8] |
|  | Alcohol cardiomyopathy | I42.6 | 100% AAF per definition |
|  | Cardiac arrhythmias | I47-I49 | Samokhvalov et al., 2010 [9] |
|  | Hemorrhagic stroke | I60-I62 | Patra et al., 2010 [10] |
|  | Ischemic stroke and other non- hemorrhagic strokes | I63-I66 | Patra et al., 2010 [10] |
| Digestive diseases | |  |  |
|  | Alcoholic gastritis | K29.2 | 100% AAF per definition |
|  | Cirrhosis of the liver | K70, K74 | Rehm et al., 2010 [11] |
|  | Acute and chronic pancreatitis | K85, K86.1 | Irving et al., 2009 [12] |
|  | Chronic pancreatitis (alcohol-induced) | K86.0 | 100% AAF per definition |
| Respiratory infections | |  |  |
|  | Lower respiratory infections | J10-J18, J20-J22 | Samokhvalov et al., 2010 [13] |
| Unintentional injuries | |  |  |
|  | Motor vehicle accidents | § | Taylor et al., 2010 [14] for relative risk, Rehm et al., 2008 [15] and Taylor et al., 2008 [16] for AAF calculation methods |
|  | Poisonings | X40-X44, X46-X49 | Taylor et al., 2010 [14] for relative risk, Rehm et al., 2008 [15] and Taylor et al., 2008 [16] for AAF calculation methods |
|  | Falls | W00-W19 | Taylor et al., 2010 [14] for relative risk, Rehm et al., 2008 [15] and Taylor et al., 2008 [16] for AAF calculation methods |
|  | Fires | X00-X09 | Taylor et al., 2010 [14] for relative risk, Rehm et al., 2008 [15] and Taylor et al., 2008 [16] for AAF calculation methods |
|  | Accidental Poisonings and exposure to alcohol | X45 | 100% AAF per definition |
|  | Drowning | W65-W74 | Taylor et al., 2010 [14] for relative risk, Rehm et al., 2008 [15] and Taylor et al., 2008 [16] for AAF calculation methods |
|  | Other Unintentional injuries | †Rest of V-series and W20-W64, W 75-W99, X10-X39, X50-X59, Y40-Y86, Y88, and Y89 | Taylor et al., 2010 [14] for relative risk, Rehm et al., 2008 [15] and Taylor et al., 2008 [16] for AAF calculation methods |
| Intentional injuries | |  | Taylor et al., 2010 [14] for relative risk, Rehm et al., 2008 [15] and Taylor et al., 2008 [16] for AAF calculation methods |
|  | Self-inflicted injuries | X60-X64, X66-X84 and Y87.0 | Taylor et al., 2010 [14] for relative risk, Rehm et al., 2008 [15] and Taylor et al., 2008 [16] for AAF calculation methods |
|  | Intentional self-poisoning by and exposure to alcohol | X65 | 100% AAF per definition |
|  | Homicide | X85-Y09, Y87.1 | Taylor et al., 2010 [14] for relative risk, Rehm et al., 2008 [15] and Taylor et al., 2008 [16] for AAF calculation methods |
|  | Other intentional injuries | Y35 | Taylor et al., 2010 [14] for relative risk, Rehm et al., 2008 [15] and Taylor et al., 2008 [16] for AAF calculation methods |
| Ethanol and methanol toxicity, undetermined intent | | Y15 | 100% AAF per definition |
| § V021–V029, V031–V039, V041–V049, V092, V093, V123–V129, V133–V139, V143–V149, V194–V196, V203–V209, V213–V219, V223–V229, V233–V239, V243–V249,V253–V259, V263–V269, V273– V279, V283–V289, V294–V299, V304–V309, V314–V319, V324–V329, V334–V339, V344–V349, V354–V359, V364–V369, V374–V379, V384–V389, V394–V399, V404–V409, V414–V419, V424–V429, V434–V439, V444–V449, V454–V459, V464– V469, V474–V479, V484–V489, V494–V499, V504–V509, V514–V519, V524–V529, V534–V539, V544–V549, V554–V559, V564–V569, V574–V579, V584–V589, V594–V599, V604–V609, V614–V619, V624–V629, V634–V639, V644–V649, V654– V659, V664–V669, V674–V679, V684–V689, V694–V699, V704–V709, V714–V719, V724–V729, V734–V739, V744–V749, V754–V759, V764–V769, V774–V779, V784–V789, V794–V799, V803–V805, V811, V821, V830–V833, V840–V843, V850– V853, V860–V863, V870–V878, V892. †Rest of V = V-series MINUS §. | | | |

Reference List

1. Lönnroth K, Williams B, Stadlin S, Jaramillo E, Dye C (2008) Alcohol use as a risk factor for tuberculosis - a systematic review. BMC Public Health 8: 289.

2. Rehm J, Samokhvalov AV, Neuman MG, Room R, Parry CD, et al. (2009) The association between alcohol use, alcohol use disorders and tuberculosis (TB). A systematic review. BMC Public Health 9: 450.

3. Baan R, Straif K, Grosse Y, Secretan B, El Ghissassi F, et al. (2007) Carcinogenicity of alcoholic beverages. Lancet Oncol 8: 292-293.

4. Corrao G, Bagnardi V, Zambon A, La Vecchia C (2004) A meta-analysis of alcohol consumption and the risk of 15 diseases. Prev Med 38: 613-619.

5. Baliunas D, Taylor B, Irving H, Roerecke M, Patra J, et al. (2009) Alcohol as a risk factor for type 2 diabetes - a systematic review and meta-analysis. Diabetes Care 32: 2123-2132.

6. Samokhvalov AV, Irving H, Mohapatra S, Rehm J (2010) Alcohol consumption, unprovoked seizures and epilepsy: a systematic review and meta-analysis. Epilepsia 51: 1177-1184.

7. Taylor B, Irving HM, Baliunas D, Roerecke M, Patra J, et al. (2009) Alcohol and hypertension: gender differences in dose-response relationships determined through systematic review and meta-analysis. Addiction 104: 1981-1990.

8. Roerecke M, Rehm J (2011) Ischemic heart disease mortality and morbidity in former drinkers: a meta-analysis. Am J Epidemiol 73: 245-258.

9. Samokhvalov AV, Irving HM, Rehm J (2010) Alcohol as a risk factor for atrial fibrillation: a systematic review and meta-analysis. Eur J Cardiovasc Prev Rehabil 17: 706-712.

10. Patra J, Taylor B, Irving H, Roerecke M, Baliunas D, et al. (2010) Alcohol consumption and the risk of morbidity and mortality from different stroke types - a systematic review and meta-analysis. BMC Public Health 10: 258.

11. Rehm J, Taylor B, Mohapatra S, Irving H, Baliunas D, et al. (2010) Alcohol as a risk factor for liver cirrhosis - a systematic review and meta-analysis. Drug Alcohol Rev 29: 437-445.

12. Irving HM, Samokhvalov A, Rehm J (2009) Alcohol as a risk factor for pancreatitis. A systematic review and meta-analysis. JOP 10: 387-392.

13. Samokhvalov AV, Irving HM, Rehm J (2010) Alcohol consumption as a risk factor for pneumonia: systematic review and meta-analysis. Epidemiol Infect 138: 1789-1795.

14. Taylor B, Irving HM, Kanteres F, Room R, Borges G, et al. (2010) The more you drink, the harder you fall: a systematic review and meta-analysis of how acute alcohol consumption and injury or collision risk increase together. Drug Alcohol Depend 110: 108-116.

15. Rehm J, Room R, Taylor B (2008) Method for moderation: measuring lifetime risk of alcohol-attributable mortality as a basis for drinking guidelines. Int J Methods Psychiatr Res 17: 141-151.

16. Taylor B, Rehm J, Room R, Patra J, Bondy S (2008) Determination of lifetime injury mortality risk in Canada in 2002 by drinking amount per occasion and number of occasions. Am J Epidemiol 168: 1119-1125.
