# Supplementary material for: Mortality and Potential Years of Life Lost Attributable to Alcohol Consumption by Race and Sex in the United States in 2005
Source: PLoS One. 2013 Jan 2;8(1):e51923. doi: 10.1371/journal.pone.0051923 (PMC3534703; doi:10.1371/journal.pone.0051923)
Supplement: Appendix S3 — Alcohol-attributable fraction modeling methodology. (DOCX) [file pone.0051923.s003.docx]

## Appendix S3: Alcohol-attributable fraction modeling methodology

*AAFs for chronic and infectious diseases, except ischemic heart disease*

AAF calculations were based on the distribution of alcohol consumption, prevalences of current drinkers, former drinkers and lifetime abstainers, and the RR as follows:

where P_abs_ represents lifetime abstainers, P_former_ is the prevalence of former drinkers, RR_former_ is the RR for former drinkers, P_current_ is the prevalence of current drinkers who consume an average daily amount (x) of alcohol, and RR_current_ is the RR given an average daily consumption of x.

*AAFs for ischemic heart disease*

Ischemic heart disease risk is impacted by both average volume of alcohol consumption and patterns of drinking [1,2]. For our modeling, we based RR on the well-known J-shaped curve [3–5], for people with at least one irregular heavy drinking occasion per month [6].

*AAFs for injuries (harms to oneself)*

The AAFs for injuries were modeled according to methodology which takes into account two dimensions of alcohol consumption:

1. binge drinking (both the number of occasions and the amount consumed per occasion), and
2. average daily alcohol consumption (on non-binge days).

When calculating the AAFs, we also included alcohol metabolism rates for men and women to calculate a person’s time at risk of an injury outcome, according to methods outlined by Taylor and colleagues [7]. The AAFs for intentional and unintentional injuries attributable to alcohol consumption were calculated as follows:

*$AAF=\frac{P_{abs}+P_{current(non-binge)}RR_{current(non-binge)}+P_{current(binge)}RR_{current(binge)}-1}{P_{abs}+P_{current(non-binge)}RR_{current(non-binge)}+P_{current(binge)}RR_{current(binge)}}$*

where P_abs_ represents the prevalence of current abstainers, and P_current(binge)_ and P_current(non-binge)_ are the prevalence of current drinkers who engage in binge drinking and the prevalence of current drinkers who do not engage in binge drinking, respectively. The RRs were calculated separately for current drinkers who engage in binge drinking and current drinkers who do not engage in binge drinking:

$RR_{\mathrm{current}\left( non-binge \right)}=\left( RR_{\mathrm{average}}-1 \right)*P_{\mathrm{nonbingedays}}+$ $RR_{\mathrm{current}\left( non-binge \right)}=\left( RR_{\mathrm{average}}-1 \right)*P_{\mathrm{nonbingedays}}+$1

and

$$RR_{current\left( binge \right)}=\left( RR_{average}-1 \right)*P_{nonbingedays}+\left( RR_{binge}-1 \right)*P_{bingedays}+1$$

where

$$RR_{average}=P_{dayatrisk}(x)*(RR_{avg\_non-binge}(x)-1)+1$$

and

$$RR_{binge}=P_{dayatrisk}(x)*\left( RR_{binge}\left( x \right)-1 \right)+1$$

In the above formulae, P_dayatrisk_ represents the proportion of a day at risk, and RR_binge_ and RR_current_ are the relative risks for injury given an amount of alcohol consumed. P_dayatrisk_ is calculated based on the average rate at which alcohol is metabolized, thus corresponding to the time during which the blood alcohol level was sufficiently elevated to increase the risk of injury.

Since these AAFs were calculated based on samples of emergency room patients, we adjusted the AAF for mortality from non-motor vehicle accidents by multiplying it by 9/4 [8], based on two studies that compared blood alcohol levels of emergency room patients with blood alcohol levels obtained from coroners’ reports of patients who died from an injury [9,10].

For women, the AAF for motor vehicle accidents was calculated by multiplying the AAF for motor vehicle accidents for men by the product of the *per capita* consumption of alcohol for women divided by the *per capita* consumption of alcohol for men. This was done as the RR function for motor vehicle accidents was considered valid only for men [11].

Reference List

1. Puddey IB, Rakic V, Dimmitt SB, Beilin LJ (1999) Influence of pattern of drinking on cardiovascular disease and cardiovascular risk factors - a review. Addiction 94: 649-663.

2. Rehm J, Sempos C, Trevisan M (2003) Average volume of alcohol consumption, patterns of drinking and risk of coronary heart disease - a review. J Cardiovasc Risk 10: 15-20.

3. Roerecke M, Rehm J (2011) Alcohol consumption and the risk for morbidity and mortality of ischemic heart disease. - A systemic review and meta-analysis. Toronto, Canada: Centre for Addiction and Mental Health.

4. Corrao G, Rubbiati L, Bagnardi V, Zambon A, Poikolainen K (2000) Alcohol and coronary heart disease: A meta-analysis. Addiction 95: 1505-1523.

5. Ronksley PE, Brien SE, Turner BJ, Mukamal KJ, Ghali WA (2011) Association of alcohol consumption with selected cardiovascular disease outcomes: a systematic review and meta-analysis. BMJ 342: d671.

6. Roerecke M, Rehm J (2010) Irregular heavy drinking occasions and risk of ischemic heart disease: a systematic review and meta-analysis. Am J Epidemiol 171: 633-644.

7. Taylor B, Shield K, Rehm J (2011) Combining best evidence: A novel method to calculate the alcohol-attributable fraction and its variance for injury mortality. BMC Public Health 11: 265.

8. Rehm J, Room R, Monteiro M, Gmel G, Graham K, et al. (2004) Alcohol Use. In: Ezzati M, Lopez AD, Rodgers A, Murray CJL, editors. Comparative quantification of health risks: global and regional burden of disease attributable to selected major risk factors. Geneva, Switzerland: World Health Organization. pp. 959-1109.

9. Cherpitel C (1994) Alcohol and casualties: a comparison of emergency room and coroner data. Alcohol Alcohol 29: 211-218.

10. Cherpitel C (1996) Alcohol in fatal and nonfatal injuries: a comparison of coroner and emergency room data from the same country. Alcohol Clin Exp Res 20: 338-342.

11. Rehm J, Shield K, Rehm M, Gmel Gj, Frick U (2012) Alcohol consumption, alcohol dependence, and attributable burden of disease: potential gains from effective interventions for alcohol dependence. Toronto, Canada: Centre for Addiction and Mental Health.
