# Supplementary material for: Mortality and Potential Years of Life Lost Attributable to Alcohol Consumption by Race and Sex in the United States in 2005
Source: PLoS One. 2013 Jan 2;8(1):e51923. doi: 10.1371/journal.pone.0051923 (PMC3534703; doi:10.1371/journal.pone.0051923)
Supplement: Appendix S4 — Alcohol-attributable fractions by major causes of death. (DOCX) [file pone.0051923.s004.docx]

## Appendix S4: Alcohol-attributable fractions by major causes of death

Figure S1. Percentage of all deaths attributable to alcohol consumption by major cause of death for men aged 15 to 64 years


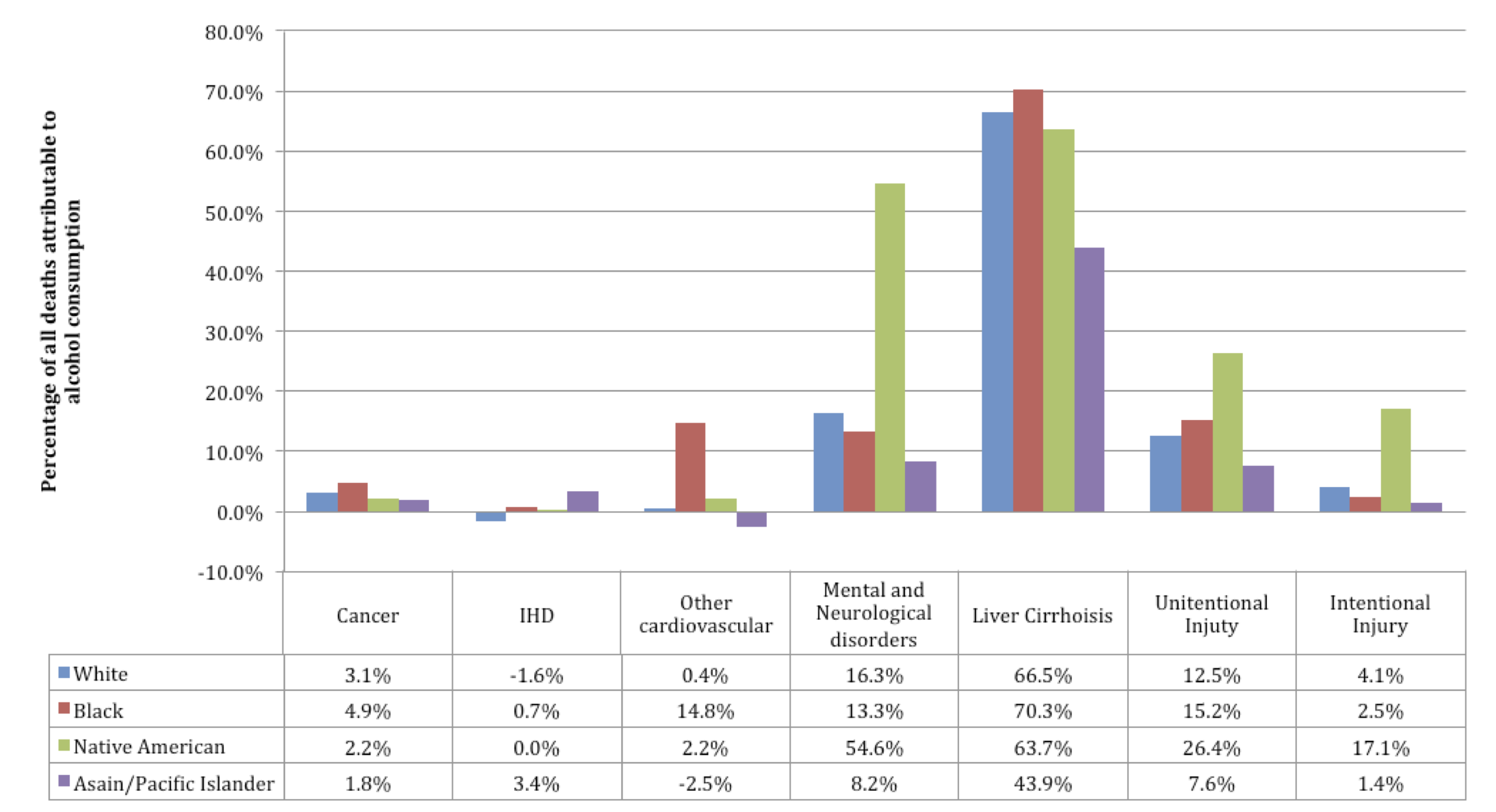


Figure S2. Percentage of all deaths attributable to alcohol consumption by major cause of death for women aged 15 to 64 years
